# Origin and Function of Bitumen-Coated Torpedo Jars from the Sasanian to Early Islamic Period Fort of Fulayj in Oman

**DOI:** 10.3390/molecules31152571

**Published:** 2026-07-23

**Authors:** Jacques Connan, Seth Priestman, Michael H. Engel, Alex Zumberge, Gazmend Elezi, Nasser S. Al-Jahwari

**Affiliations:** 1 Institute of Chemistry, Université de Strasbourg, 23 rue Antoine de Saint-Exupéry, 64000 Pau, France; 2 Institute for the Study of Ancient Cultures, University of Chicago, 1155 E 58th St., Chicago, IL 60637, USA; t-9pries@uchicago.edu; 3 School of Geosciences, The University of Oklahoma, 100 Esat Boyd Street, Norman, OK 73019, USA; ab1615@ou.edu; 4 GeoMark Research Inc., 9748 Whithorn Drive, Houston, TX 77095, USA; azumberge@geomarkresearch.com; 5 Pasarow Mass Spectrometry Laboratory, University of California Los Angeles, 760 Westwood Plaza, Los Angeles, CA 90024, USA; gazelezi@ucla.edu; 6 Cotsen Institute of Archaeology, University of California Los Angeles, 308 Charles E Young Drive North, Los Angeles, CA 90024, USA; 7 Department of Archaeology, Sultan Qaboos University, Al Seeb Al Khoudh, Muscat OM 123, Oman; jahwari@squ.du.om

**Keywords:** bitumen, torpedo jar, Sasanian, Fulayj, Oman, steranes, terpanes, isotopes (C, H), biodegradation, natural asphalts, Iran, red wine

## Abstract

Geochemical and isotopic analysis of bitumen-lined transport container vessels, so called ‘torpedo jars’, from the Sasanian to Early Islamic period fort of Fulayj in Oman confirms the presence of two distinct compositional categories that can be matched to contemporary sources in different areas of southwest Iran. It appears that the bitumen used to line jars was extracted from different geographic areas, hinting at the existence of multiple production locations for this vessel class. In terms of the function of the jars, organic residue analysis provides a positive identification of biomarkers associated with the storage and transportation of red wine. These results provide the first published confirmation of the long-suspected association between torpedo jars and wine transportation from the core urbanised zone of the Sasanian and Early Islamic imperial heartlands towards the Persian Gulf and Indian Ocean maritime sphere. The results have added significance as the samples are derived from relatively accurately dated archaeological contexts spanning the period before and after the Islamic conquest.

## 1. Introduction

A sample of 15 typical and representative sherds of bitumen-coated torpedo jars have been selected for analysis from the Late Sasanian to Early Islamic period fort of Fulayj in Oman [1,2]. This is one of the few sites in Oman securely dated to the period. Torpedo jars represent a dominant class of ceramic material within the assemblage and a common component of most contemporary sites within the region. They are a distinctive class of handless transport container vessels with a narrow mouth and a pointed base coated with a waterproof bitumen lining. They are found very widely distributed across the Mesopotamian plain, southern Iran and along the Persian Gulf and western Indian Ocean littoral. Isolated examples are also attested from shipwreck assemblages in Southeast Asia [3,4]. Torpedo jars represent the functional equivalent of the Mediterranean amphorae within the Indian Ocean. By analogy, this vessel class potentially holds the key to unlocking a significant part of the economic history of the Late Antiquity period across much of Western Asia. As such, they deserve a high degree of scientific focus and attention. Despite a concerted attempt over the past two decades to better define the typology, provenance and dating of the class [3,5,6,7,8,9,10,11,12,13,14,15,16,17,18], it remains an inconvenient fact that their chronology is broad, spanning the period from at least the 3rd to 9th c. CE and with little obvious stylistic, morphological or technical change during this long period of circulation. Recent research on the composition of both the ceramic fabric and bitumen lining [3,12,15,17,18] continues to push at the boundaries of what is admittedly a complex archaeological dataset. From previous work, we have successfully established that the production of this class occurred within the general area of southern Iraq and/or southwest Iran. The potential to further refine our knowledge of the production location(s) of this class remains open to investigation. The current contribution adds to this body of research by analysing samples from securely dated contexts from the fort of Fulayj in Oman. Fulayj is itself significant as the first clearly attested site containing a Sasanian military presence facing directly onto the Indian Ocean [1].

The aim of our paper is to address several questions:

1. Can the bituminous residues in the interior faces of sherds be geochemically characterised and their sources more closely identified?
2. What does the provenance data imply about the procurement and trade networks of bitumen?
3. Is it possible to identify the nature of the contents transported or stored in the torpedo jars?

## 2. Archaeological Context

**Figure 1:**
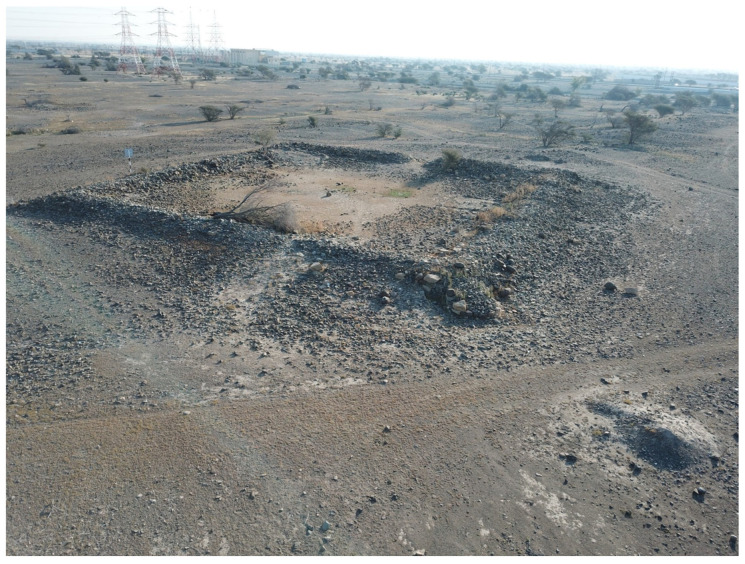
Aerial photography of the Fulayj fort.

Fulayj (Figure 1) is the site of a small c. 30 × 30 m, heavily defended, square fortification with projecting ‘U’-shaped corner and entrance flanking towers and a single entranceway facing to the east. It was built with a thick, neatly constructed stone base most likely supporting a much more substantial mudbrick superstructure. Radiocarbon dating indicates that the fort was built in the late pre-Islamic period sometime between the early 5th and mid-6th c. CE [2]. The latest results of excavation and dating indicate that there was a brief horizon of related activity in the same location preceding the fort construction [2]. This may represent the fort construction horizon, or there may have been an ephemeral defence and encampment established before the construction of the main military monument. These considerations are set out elsewhere. The location of the site is c. 30 km southwest of the major Indian Ocean emporium of Sohar, which may already have attained some prominence before the Islamic conquest [19,20] though that point is subject to on-going investigation [21,22]. The dating of the fort, its clear architectural links with similar small Late Antique fortlets across the Middle East, and its expert construction all strongly suggest that it was built by an external military force, which we can associate historically with the projection of Sasanian imperial power and the settlement of a Persian population on the al-Batinah during the Late Sasanian period [1]. As such, Fulayj represents the first securely identified evidence of a Sasanian military presence in Oman. Elsewhere, there has been much discussion over the extent of Sasanian involvement in the region. A positive reading of the meagre historical information has been used to argue for the settlement and control of the al-Batinah by a Persian population and the wide-scale economic development of the area in the late pre-Islamic period [23]. More recent analysis suggests that the historical interpretation needs to be treated with more caution [24,25]. The limited scale of archaeological discoveries connected with Sasanian activity in Arabia has also called into question the hypothesis of this southern territorial expansion. The discovery of a small fort at Fulyj marks an important addition to our information, yet the interpretation of this, so far, single isolated structure is not straightforward. Was it part of a larger chain of forts protecting the coastal region, or perhaps the first step in the construction of a defensive system that was never completed? Close analysis of the associated artefactual assemblage potentially enriches our understanding of its use and relationship with the wider imperial structure of the Sasanian empire. A further telling consideration is whether the occupation of Fulayj was short- or long-lived. Finds within the fort are generally scarce.

Significantly, the building underwent certain modifications and continued to be occupied during the first century following the Islamic conquest [26]. At this time mudbrick rooms were inserted into one corner of the fort, and the defensive aspect of the gateway was modified. It is possible that the site took on a less overtly military function.

Torpedo jars form a significant category of ceramic imports within the assemblage from Fulayj. They are represented through every part of the occupation, including both its late pre-Islamic foundation and the reuse of the building during the seventh century in the period following the Islamic conquest of Eastern Arabia. In general, the quantity of finds recovered from all parts of the excavations is relatively limited. If one disregards the large number of sherds from an earlier unrelated occupation (in the Iron Age) and some later ephemeral use of the area during the Late Islamic period, a total of 1230 sherds were recovered that are directly associated with the occupation of the fort. Of these, 117 sherds are torpedo jars (9.5% of the pottery). Despite our very intensive mapping of the finds, involving the 3D location of each sherd across excavations covering around 25% of the interior of the structure, no significant zonal patterning could be observed. The sherds appear to be scattered according to a process of secondary discard and general loss with a progressive drop-off distribution away from the structure. Rather than a spatial location, the important consideration from the perspective of our analysis here is the stratigraphic position of sherds within the sequence. With the possibility of upward migration through residuality, the phasing broadly relates to several significant horizons of site use (see more below). It should be noted that torpedo jars from Fulayj show no significant macroscopic variation. The samples that were selected are typical of the assemblage as a whole. The general sparsity of finds within the fort suggests either that the occupation of the fort was short-lived or that the intensity of domestic activity was limited.

## 3. Materials and Methods

### 3.1. Samples

**Figure 2:**
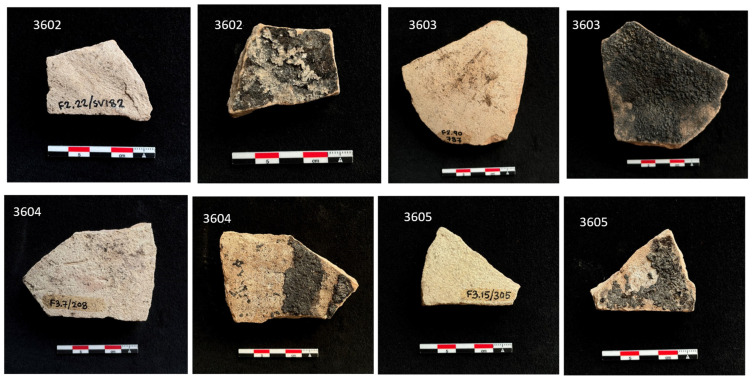
Examples of four of the samples from Fulayj showing various aspects of the bitumen coating the interior face of potsherds.

Fifteen samples of torpedo jars (Table 1) were selected (12.8% of all torpedo jar sherds). The bitumen coating from the interior was isolated by scraping the surface with a scalpel. The spatial location of the samples within the structure does not appear to relate in a significant way to the zonal concentration of activity within the fort and is therefore not considered further. Instead, we will present the assemblage according to stratigraphic phase. Four of the samples are illustrated in Figure 2. They present examples of different situations with variable crusts of bitumen. No bitumen occurs on the exterior face of the sherds.

### 3.2. Analytical Procedures

#### 3.2.1. Bitumen Analysis

All archaeological and oil seep samples used as references were subjected to the same analytical procedure conducted at GeoMark Research Ltd. (Houston, TX, USA). The dichloromethane–methanol (93:3) extract was deasphalted using *n*-hexane. The desasphalted fraction was separated into saturated hydrocarbons, aromatic hydrocarbons and resins using gravity flow column chromatography employing a 100–200 mesh silica gel support activated at 400 °C prior to use. Hexane was used to elute saturates, dichloromethane was used to elute the aromatic hydrocarbons and dichloromethane/methanol (50:50) was used to elute the resin (NSO) fraction. Following solvent evaporation, the recovered fractions were quantified gravimetrically. The C_15+_saturated hydrocarbon fraction was subjected to molecular sieve filtration (Union Carbide S-115 powder) after the technique described by West et al. [27]. An aliquot of the total alkane fraction was not fractionated by silicalite in order to preserve access to the *n*-alkanes.

GC-MS of the C_15+_branched and cyclic hydrocarbon fractions was performed using an Agilent 7890A (split injection) interfaced to an Agilent 5975C mass spectrometer (Wilmington, DE, USA). The HP-2 column (50 m × 0.2 mm, 0.11 µm film thickness) was temperature-programmed from 150 °C to 325 °C at 2 °C/minute and held for 10 min. The mass spectrometer was run in the selected ion mode (SIM), monitoring ions *m*/*z* 177, 191, 205, 217, 218, 231 and 253 amu for branched and cyclic alkanes. The quantitative data were produced using peak surface.

To determine the absolute concentration of individual biomarkers, a deuterated internal standard (d4-C_29_ααα20Rsterane, Chiron lab, Norway) was added to the C_15+_branched/cyclic hydrocarbon fraction. Response factors (RFs) at 221 for the deuterated standard to hopane (*m*/*z* 191) and sterane (*m*/*z* 217) authentic standards were found to be 1.4 for terpanes and 1.0 for steranes. Concentrations of individual biomarkers were determined using the following equation: Conc. (in ppm) = (Ht biomarker) (ng standard)/(Ht standard) (RF) (mg B/C fraction).

The C_15+_saturates, C_15+_aromatics, asphaltenes and resins were analysed for their respective carbon isotope (δ^13^C in ‰/VPDB) compositions. Approximately 200–300 μg of each sample was loaded and sealed in a tin cup (Costech, Valencia, CA, USA). Samples were placed in sequence in an autosampler mounted on a Costech elemental analyser interfaced through a Conflo III valve with a Thermo Delta V Plus isotope ratio mass spectrometer (Thermo Fisher Scientific, West Palm Beach, FL, USA). The δ^13^C values are reported in per mil (‰) relative to the Vienna Pee Dee Belemnite (VPDB standard, uncertainty ± 0.1‰). The procedure for the stable hydrogen isotope analysis of the resin and asphaltene fractions is identical to that recently reported by [12].

#### 3.2.2. Wine Detection

Organic mass spectrometry has been successfully applied to characterise paleomolecules associated with wine residues in archaeological ceramics. Targeted and nontargeted methods have utilised liquid chromatography-tandem mass spectrometry (LC-MS/MS) and gas chromatography-mass spectrometry (GC-MS), focusing on organic acids and phenols [28,29,30,31]. Despite success, confidence in analytical results is often limited, especially when the artefacts under analysis lack an explicit archaeological context. One of the main issues is the inability to attribute the detected molecules exclusively to wine, given their presence in both fresh and fermented grapes. Thus, without additional information from the archaeological context, detecting ancient wine through residue analysis remains ambiguous [32]. To address these issues, our research focused on new wine biomarkers, which are water-soluble molecules naturally synthesised during grape fermentation and are responsible for the colour of wine [33,34,35].

##### Analysis of Wine Residues

The analysis of wine residues employs a new analytical technique developed by Gazmend Elezi at the UCLA Pasarow Mass Spectrometry Laboratory to target ancient wine biomarkers.

A liquid chromatography-tandem mass spectrometry (LC-MS/MS) assay was developed to detect pyranoanthocyanins present in red wine such as Vitisin A (VA) and Vitisin B (VB). The method was developed and evaluated using modern red wine and appropriate positive and negative controls before being applied to archaeological materials. The details of the new method have been submitted [36]. The analytical method was designed as a qualitative LC-MS/MS approach for the detection of wine-specific biomarkers in archaeological ceramics. The objective was to determine the presence or absence of diagnostic pyranoanthocyanins based on their chromatographic retention times and characteristic MS/MS fragmentation patterns rather than to perform absolute quantification. Conclusive results for the detection of pyranoanthocyanins in archaeological ceramics, using this method in another study, were recently published [37]. The sampling method for archaeological ceramics was designed to include fragments with intact interior surfaces coated with bitumen. Previous experimental studies have shown that wine molecules are present in the ceramic fabric, albeit at low concentrations, even when tar layers are applied on the interior surface [38,39]. Roman amphorae (Dressel 2–4), coated with Pinaceae products which had undergone different stages of preparation and alteration, confirm wine markers in all the samples [39]. The wine seeps into the bitumen itself or penetrates through fissures into the ceramic body.

##### Sample Preparation

The archaeological samples used in the detection of organic residues were pulverised in a pestle and mortar, and 2 g of pottery powder was treated with 5 mL of a methanol/DI water/Formic Acid mixture 50:50:0.1 (*v*/*v*/*v*) in a glass test tube. The sample size was determined by the limited availability of archaeological material. The samples were vortexed and centrifuged at 2000× *g* for 15 min, and the supernatants were transferred into new tubes. The extraction procedure was repeated by adding 3 mL of the mixture, and the supernatants were transferred into the new tubes. One hundred picomoles of daidzin dissolved in ethanol were added as a process internal standard. Daidzin was selected because it possesses structural and physicochemical characteristics similar to those of the target pyranoanthocyanin biomarkers yet remains chromatographically distinct. This minimises analytical interference while providing comparable extraction, chromatographic and ionisation behaviour. The internal standard was therefore used to monitor sample preparation, extraction efficiency, analytical recovery and instrument performance throughout the analytical workflow. The samples were dried in a vacuum concentrator for 4 h. The dried samples were resuspended in 100 µL of methanol/water/Formic Acid (95:5:01) (*v*/*v*/*v*), vortexed thoroughly and centrifuged at 2000× *g* for 5 min. The supernatant was transferred to HPLC polypropylene vials, and 25 µL was injected for analysis.

##### Liquid Chromatography–Tandem Mass Spectrometry LC-MS

A targeted liquid chromatography-tandem mass spectrometry (LC-MS/MS) assay was developed on a Linear Ion Trap LTQ-XL (Thermo Scientific, Walyham, USA) mass spectrometer coupled to a Dionex Ultimate 300 HPLC system (Thermo Scientific) through a reversed-phase GL Science analytical column (Inert Sustain 2 µm, Phenyl 150 × 2.1 mm). The instrument was optimised in positive-ion mode monitoring transitions for Malvidine 3-glucoside (Mv3g) 493–331 *m*/*z*, Vitisin A (VA) 561–399 *m*/*z*, Vitisin B (VB) 517–355 *m*/*z* and Daidzin (DA) 417–255 *m*/*z*. The mobile phase consisted of A (99.9:0.1 *v*/*v* water/Formic Acid) and B (99.9:0.1 *v*/*v* Acetonitrile/Formic Acid). The HPLC method utilised the linear gradient mixture of eluents A and B to elute the targeted compounds (min/%B: 0/5, 5/5, 12/40, 26/75, 28/5, 40/5). The *m*/*z* of a fragment ion from each compound was monitored at a specific LC retention time to ensure their specificity, reproducibility and accurate identification.

The method evaluation included analyses of modern red wine, blank ceramic extracts and ceramic samples spiked with red wine, which served as positive and negative controls to verify successful extraction and specific detection of the target biomarkers. Spike-recovery experiments performed using ceramic samples fortified with red wine demonstrated recoveries of approximately 70–80%, supporting the efficiency of the extraction protocol.

## 4. Results

### 4.1. Gross Composition

Gross composition data for the analysed bitumens are listed in Table 2. Extractable organic matter in dichloromethane (EO% by weight/sample), which represents the amount of bitumen, ranges between 9.8 and 51% by weight with an average value of 37.9%. All samples are quite rich in bitumen.

**Figure 3:**
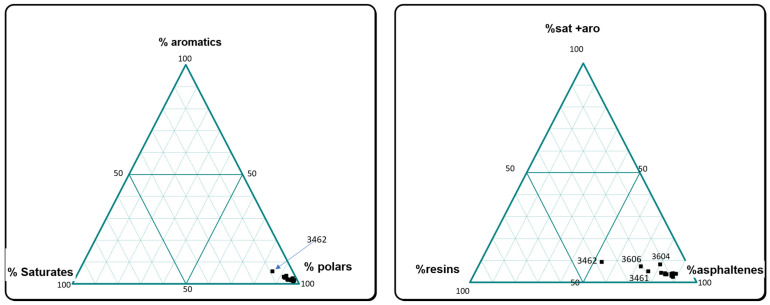
Gross composition of the dichloromethane extract in two ternary diagrams: %saturates vs. %aromatics vs. %polars and %sat + aro vs. %resins vs. %asphaltenes.

A plot of %sat (saturated hydrocarbons) vs. %aro (aromatic hydrocarbons) vs. %polars (=resins + asphaltenes) in Figure 3 shows that most samples are extremely rich in polars. One sample (No. 3462) is slightly different in the enrichment in hydrocarbons (9.3%).

A plot of %sat + aro vs. %resins (NSO’s) vs. %asphaltenes shows a diversified situation with samples extremely rich in asphaltenes (more than 80%) and some others (No. 3462, 3606 and 3461) in which resins are higher, between 18.6 and 37.1%. The compositions of archaeological samples characterised by an enrichment in asphaltenes have been reported in many publications [40,41,42,43,44,45,46,47,48,49,50,51,52,53,54].

### 4.2. Isotope Data

**Figure 4:**
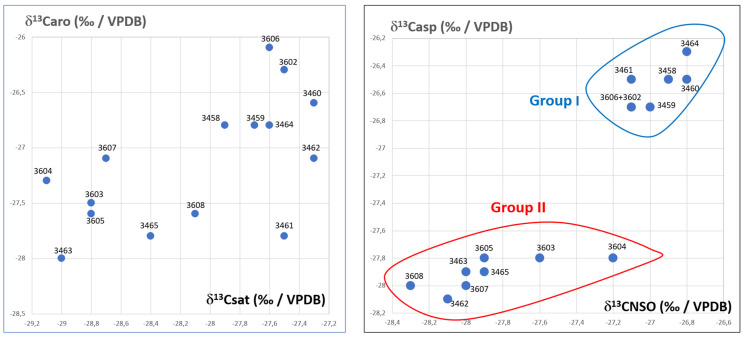
Lot of δ^13^C (‰/VPDB) of aromatics vs. δ^13^C (‰/VPDB) of saturates and δ^13^C (‰/VPDB) of asphaltenes vs. δ^13^C (‰/VPDB) of NSO for the Fulayj samples.

Carbon and hydrogen isotopes are presented in Table 2. The plot of δ^13^Csat vs. δ^13^Caro and δ^13^CNSO vs. δ^13^Casp in Figure 4 clearly shows that there are at least two distinct groups of bitumen: group one with δ^13^Casp between −27.1 and −26.8‰/VPDB and group two with δ^13^Casp between −28.3 and −27.2‰/VPDB.

**Figure 5:**
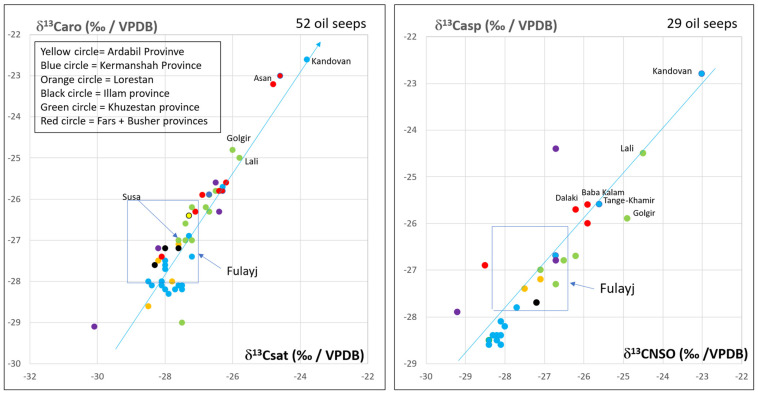
Plot of δ^13^C (‰/VPDB) of aromatics vs. δ^13^C (‰/VPDB) of saturates and δ^13^C (‰/VPDB) of asphaltenes vs. δ^13^C of NSO for the oil seeps of Iran: Comparison with Fulayj’s data.

**Figure 6:**
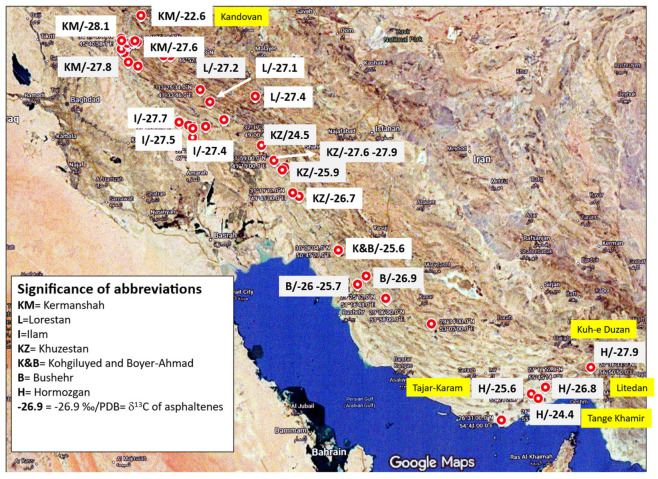
Plot of δ^13^C of asphaltenes from oil seeps on a map of Iran.

**Figure 7:**
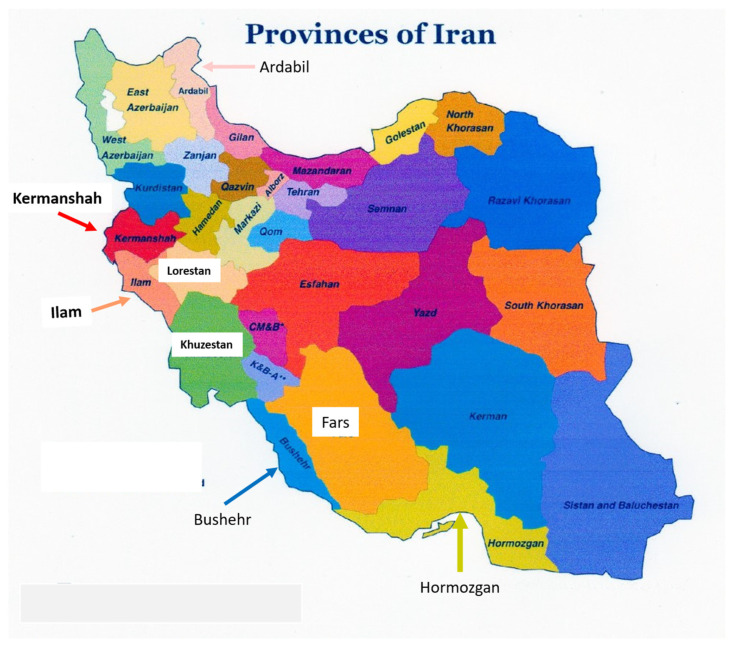
Provinces of Iran (after Nemati et al., 2018 [55]).

Comparison of these values with data acquired on oil seeps across the Middle East (Figure 5 and Figure 6) confirms the occurrence of two main groups of samples originating from different geographical locations within Iran (Figure 7). Group I corresponds to samples originating mainly from Khuzestan, Kohgiluyed and Bushehr, whereas Group II corresponds to a composition distributed in Kermanshah, Ilam and Lorestan. The oil seep of Kuh-e Duzan from Hormozgan province also corresponds to Group I. Similar situations were recorded in torpedo jars from the Phanom-Surin shipwreck in Thailand [3] and recently in torpedo jars from the al-Qusur Monastery in Kuwait [18] and other sites from Sri Lanka, India and Oman. One may notice that extreme values have never been found in the bitumen of torpedo jars associated with the Kadovan oil seep (−22.6) in Kermanshah and Tange Khamir (−24.4) in Hormozgan (Figure 7).

**Figure 8:**
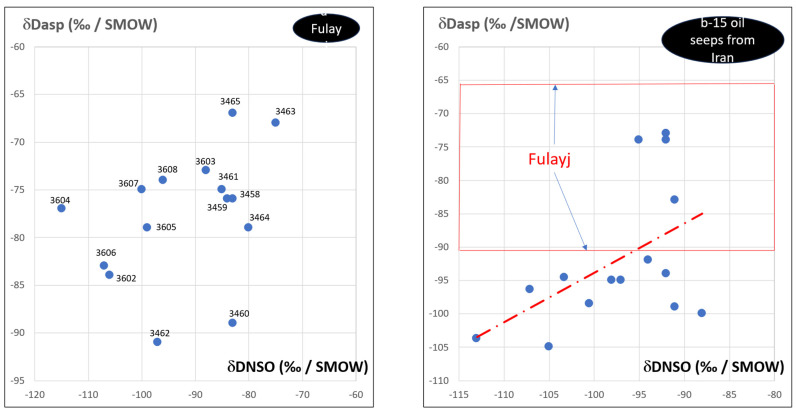
Plot of the δD (‰/VSMOW) of asphaltenes vs. δD (‰/VSMOW) of NSO. (**Left**) Data on Fulayj. (**Right**) Data on 15 oil seeps from Iran.

The plot of δDasp vs. δDNSO in Figure 8 does not show any relationship between δD values and their group, defined by their carbon isotope ratios. This feature is not surprising for these parameters are not source-dependant but rather reflect oxidation intensities among bitumens [56]. Comparison of Fulayj data (Figure 8a) with those of 15 oil seeps from Iran (Figure 8b) shows that the Fulayj asphaltenes are generally more oxidised than those of oil seeps. Resins (NSO) of Fulayj are comparable to those of oil seeps from Iran.

### 4.3. Steranes and Terpanes

**Figure 9:**
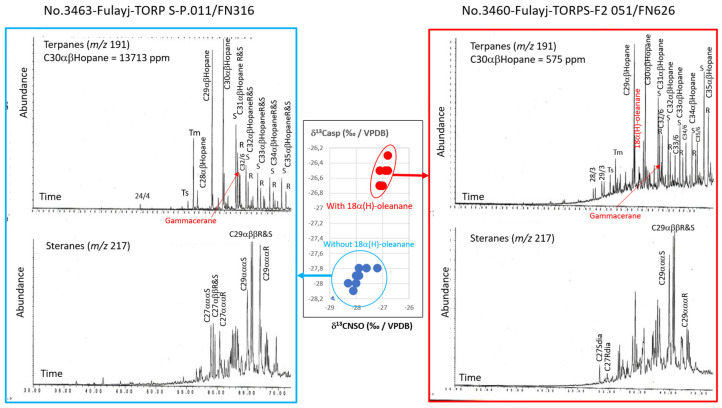
Extracted ion chromatograms of steranes (*m*/*z* 217) and terpanes (*m*/*z* 191) with the plot of δ^13^Casp vs. δ^13^CNSO: comparison of the sample No. 3643 without 18α(H)-oleanane to the sample No. 3460 with 18α(H)-oleanane.

**Figure 10:**
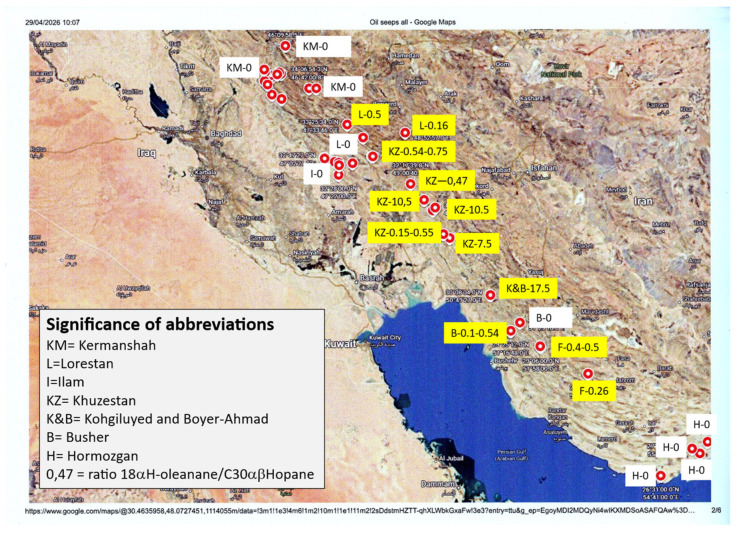
Occurrence of 18α(H)-oleanane in oil seeps from various provinces of Iran.

Steranes and terpanes were used as biomarkers to characterise the source of bitumen. Extracted ion chromatograms of terpanes (*m*/*z* 191) and steranes (*m*/*z* 217) of two archaeological samples (No. 3463 and No. 3460) are reproduced in Figure 9. Terpanes of sample No. 3463 show a complete pattern of the hopane family (C_28_αβH to C_35_αβH) with a high Tm/Ts ratio, rather high gammacerane, and almost no tricyclopolyprenanes. Their steranes are dominated by regular steranes which are slightly biodegraded. Terpanes from sample No. 3460 also show the complete hopane family with the occurrence of tricyclopolyprenanes (28/3 and 29/3), low gammacerane and the occurrence of 18α(H)-oleanane, which reflects the contribution of the Pabdeh Tertiary formation in the source rocks which have produced this type of bitumen [57]. It should be noticed that the occurrence of 18α(H)-oleanane matches samples of Group I, having δ^13^C of asphaltenes between −26 and −27‰/VPDB (Figure 4). A key point revealed by these results is the occurrence of 18α(H)-oleanane in the bitumen of Fulayj, which indicates the Iranian origin of these oils, for 18α(H)-oleanane does not exist in oil seeps from Iraq, Turkey, Israel, Syria, Kuwait, Bahrain or Oman [58,59,60,61,62,63,64]. The plot of the 18α(H)-oleanane/C30 αβHopane ratio in Figure 10 shows that the oil seeps without 18α(H)-oleanane are located in Kermanshah, Illam and Lorestan but also in Hormozgan provinces, whereas the oil seeps with 18α(H)-oleanane are located in Lorestan, Khuzestan, Bushehr and Fars provinces. It is worth noting that the oil seeps of Hormozgan provinces contain no 18α(H)-oleanane associated with δ^13^C of asphaltenes between −24.4 and −27.9‰/VPDB. This feature fully justifies the geochemical approach applied herein and in all previous geochemical studies of archaeological bitumen. The geochemical signature related to the origin of the bitumen cannot be assessed by using one technique: isotopic data acquired on chromatographic fractions must be compared to biomarker data to reach reasonable conclusions. In addition, isotopic data cannot be acquired on the raw bitumen as done by Schwartz et al. [44,50] because of the occurrence of minerals. The demonstration of the effect of carbonates upon isotopic values of the raw material has been published using Mari (Syria) samples [45].

Biomarker data are listed in Table 3.

**Figure 11:**
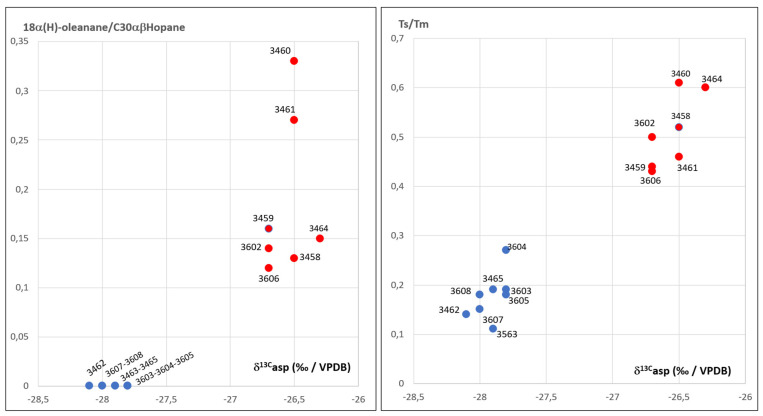
Lot of 18α(H)-oleanane/C30αβHopane vs. δ^13^Casp (‰/VPDB) and Ts/Tm vs. δ^13^Casp (‰/VPDB) with the Fulayj samples.

**Figure 12:**
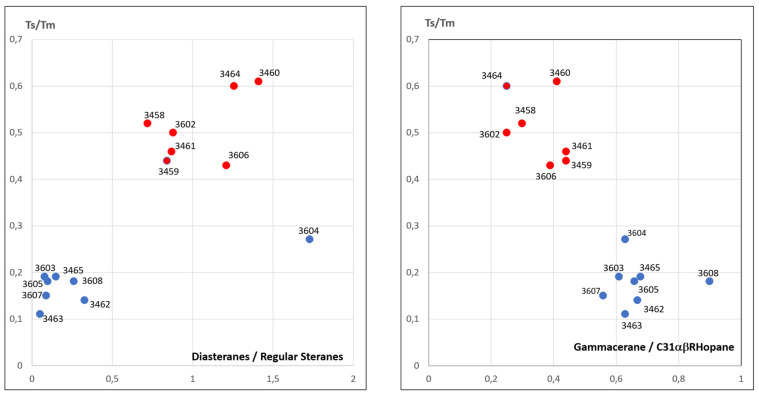
Plot of Ts/Tm vs. Diasteranes/Regular steranes and Ts/Tm vs. Gammacerane/C31αβRHopane with the samples of Fulayj.

The plot of some characteristic ratios, namely Ts/Tm vs. δ^13^C of asphaltenes (Figure 11), Ts/Tm vs. Diasteranes/Regular steranes and Ts/Tm vs. Gammacerane/C_31_αβRHopane (Figure 12), confirms the occurrence of at least two bitumen families. The first one (Group I: Nos. 3458, 3459, 3460, 3461, 3464, 3602 and 3606) corresponds to bitumen containing 18α(H)-oleanane, high diasteranes/regular steranes, high Ts/Tm ratio, low gammacerane/C_31_αβRHopane and the occurrence of tricyclic terpanes. This family was generated from a source rock (likely the Eocene–Oligocene Pabdeh formation) in which the organic matter has a vegetal contribution and was deposited in a marine environment under anaerobic conditions [57]. These bitumens are likely to originate from the Khuzestan, Kohgiluyed and Fars provinces. The second family (Group II: Nos 3462, 3463, 3465, 3603, 3604, 3605, 3607, 3608) has a low Ts/Tm ratio, mainly regular steranes and high gammacerane/C_31_αβRhopane, and likely originates from the Albian-Aptian Khazdumi formation deposited in a very anoxic hypersaline marine environment [65,66]. These archaeological bitumens came from Kermanshah, Illam and Lorestan.

**Figure 13:**
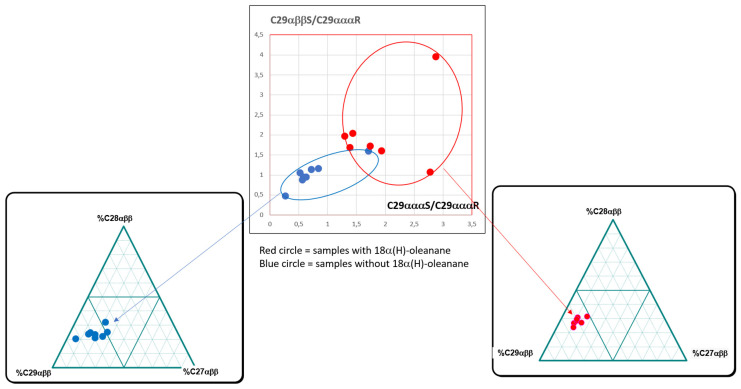
Plot of αββsteranes in a ternary diagram: %C27 vs. %C28 vs. %C29 and plot of C29αββS/C29αααR vs. C29αααS/C29αααR; comparison of samples with (red circle) and without 18α(H)-oleanane (blue circle).

A plot of data on steranes in Figure 13 differentiates the two groups of samples again. Samples with 18α(H)-oleanane are more biodegraded than samples without 18α(H)-oleanane, as indicated by the reduced C_27_ steranes. Samples without 18α(H)-oleanane have fewer C_28_ steranes, as is often the case in samples with high gammacerane which display a V-pattern (C_28_ < C_27_ and C_29_). Interestingly, samples with 18α(H)-oleanane show the selective removal of the C_29_αααR sterane, a characteristic biodegradation effect seen elsewhere in archaeological samples, for example at Kuriki Höyük in Turkey [60], Qala’t al Bahrain and Saar in Bahrain [59], Hummal in Syria [67] and Anuradhapura in Sri Lanka [56]. This selective biodegradation of the biological configuration of αααsteranes has been observed in crude oils at depth [68] and has been reproduced in 15 days under laboratory conditions using Gram-positive strains belonging to *Nocardia* and *Arthrobacter genera* [69,70].

### 4.4. Identification of Wine

**Figure 14:**
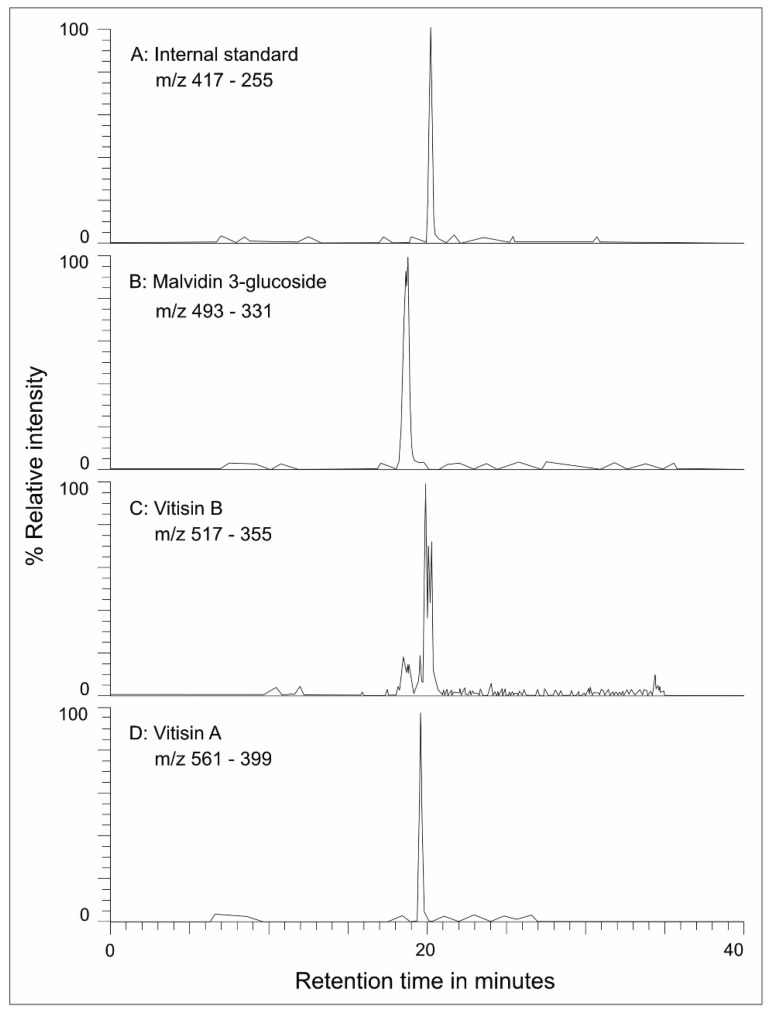
LC-MS/MS reference chromatogram of the commercial analytes depicting the signals of the monitored precursor–product ion transitions for the targeted compounds and the internal standard.

In an ongoing research program conducted by Gazmend Élezi at the UCLA Pasarow Mass Spectrometry Laboratory, a targeted LC-MS/MS assay was developed to detect wine residues [36,71]. The method targeted wine biomarkers such as Malvidine 3-glucoside (Mv3g), Vitisin A (VA) and Vitisin B (VB), monitoring transitions from precursors (Mv3g 493 *m*/*z*, VA 561 *m*/*z*, VB 517 *m*/*z*) to product ions (OE, 331 *m*/*z,* VA 339 *m*/*z,* VB 355 *m*/*z*) (Figure 14). These molecules are characteristic of red wine [33,35].

**Figure 15:**
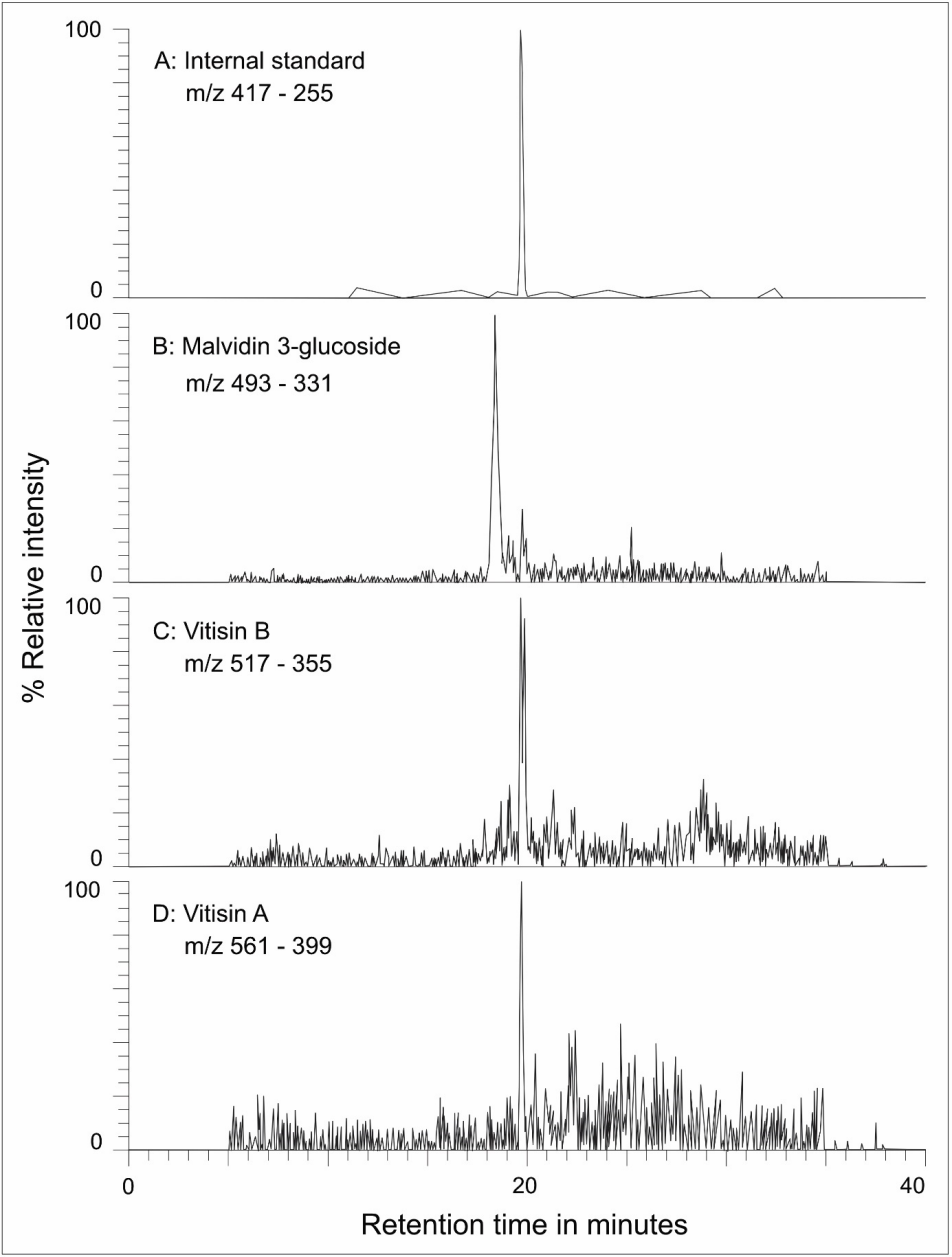
LC-MS/MS chromatogram of the archaeological sample No. 3464 depicting the signals of the monitored precursor–product ion transitions for the targeted compounds and the internal standard.

Positive readings were recorded for five samples: three (Nos 3459, 3460 and 3464) with bitumen containing 18α(H)-oleanane and two (Nos 3453 and 3465) with bitumen without 18α(H)-oleanane (Table 4, Figure 15). Sample No. 3459 comes from within a room securely dated to the 7th c. CE, seemingly to directly follow the Islamic conquest of Oman. Of course, the sherd may be residual, but the finding is at least suggestive.

## 5. Discussion

Fifteen samples of bitumen taken from torpedo jars have revealed two main compositional groups originating in southwest Iran, one from the Kermanshah-Illam-Lorestan provinces and the other from the Khuzestan-Bushehr-Fars provinces. The same situation has been documented in some other archaeological sites: al-Qusur (7th–9th c. CE) [18] and Akkaz (1st c. BCE–6th c. CE) [72] in Kuwait, Sir Bani Yas in Abu Dhabi [9] and recently in Siniya in Umm al Qaiwan [unpublished results].

Five of the samples were identified as wine containers based on specific biomarkers associated with red wine. The interpretation of this relatively small sample is complicated. Both bitumen composition groups are associated with the detected traces of wine. This suggests a consistent function regardless of the location of production. Of course, an important caveat here is that the origin of the bitumen and location of pottery production may not necessarily have been the same. Chronologically, the samples are associated with different parts of the occupation sequence (Table 1), the dating of which is constrained by radiocarbon dating evidence. Not all of this is fully published, but the outline can be summarised. One of the sherds that provided a positive identification for the presence of wine comes from Phase 2a (No. 3460). This is a thin but particularly rich occupation horizon that predates the construction of the fort. The absolute dating for this horizon is broad but delimited within the Sasanian period from the c. mid-3rd to mid-6th c. CE. It would be surprising if the dating is much earlier than the construction of the fort. A second fragment (No. 3465) belongs to Phase 2c, which comprises the ephemeral occupation directly connected with the use of the fort sometime between the early 5th to mid-6th c. CE. A third sample (No. 3459) belongs to the re-occupation of the fort and the modification of its interior with the insertion of a domestic mudbrick building in Phase 3, which can be dated between the late 6th to late 7th c. CE, certainly covering the first decades of the Islamic transition. The remaining samples are associated with the gradual collapse of the fortification, and we assume that these fragments are residual from any part of the earlier occupation. In terms of differing sources of bitumen composition, there does not appear to be any strong chronological correlation. One should be cautious in drawing any firm conclusions from this limited dataset. Although controlled burial-ageing experiments would provide additional information regarding degradation pathways and biomarker preservation, they cannot fully reproduce the complex physicochemical processes occurring over archaeological timescales. Consequently, the interpretation presented here relies on the analysis of authentic archaeological materials recovered from well-characterised archaeological contexts. The detection of wine biomarkers, considered together with the geochemical characterisation of the bitumen and the archaeological evidence, provides converging lines of evidence supporting the interpretation of these vessels while also identifying priorities for future methodological development. What the results do seem to point to is both a consistency in the pattern of usage of this vessel class through time, and to the enduring acquisition of bitumen from multiple sources. This might plausibly be connected with alternative manufacturing centres and the distribution of different regional varieties of wine as outlined in the contemporary historiography. These features require further systematic investigation at scale.

## 6. Conclusions

The coating of torpedo jars from Fulayj fort has been identified as bitumen. These bitumens originate from different provinces of Iran. Those containing 18α(H)-oleanane appear to originate from Khuzestan-Bushehr-Fars provinces. Those where 18α(H)-oleanane is absent most likely come from Kermanshah-Illam-Lorestan provinces.

Five samples spread across the two bitumen groups contain vitisin B, a biomarker characteristic of red wine. This wine was likely transported in the torpedo jars and was produced in the region from which the vessels originate. One sample dated between the late 6th–7th c. CE, suggests that the consumption of wine in the fort may have continued into the Early Islamic period.

The results indicate that throughout the occupation of the site, the fort was supplied with wine storage containers via the mechanism of long-distance maritime exchange. We might assume that this was for consumption by the inhabitants of the fort. The route of supply originates within the core territory of the Sasanian empire heartlands and involved passage through the length of the Persian Gulf and Strait of Hormuz reaching to the al-Batinah coast of Oman. It provides further evidence for the strategic integration of a remote centre of Sasanian defence into a broader regional supply network. Further research integrating additional archaeological assemblages with expanded analytical validation will further refine our understanding of biomarker preservation, vessel function and long-distance exchange networks.

## Figures and Tables

**Table 1:** Basic information on the samples.

Archaeological Reference	Phase	Date Range	Wine Biomarkers	Bitumen Source	Lab Number
F2.041/FN405	2a	Mid-3rd–mid-6thC		Khuzestan + Bushehr + Fars	**3458**
F2.051/FN626	2a	Mid-3rd–mid-6thC	Yes	Khuzestan + Bushehr + Fars	**3460**
F2.059/FN596	2a	Mid-3rd–mid-6thC		Khuzestan + Bushehr + Fars	**3461**
F2.059/FN610	2a	Mid-3rd–mid-6thC		Kermanshah + Ilam + Lorestan	**3462**
F3.022/FN475	2a	Mid-3rd–mid-6thC		Khuzestan + Bushehr + Fars	**3606**
F3.023/FN512	2a	Mid-3rd–mid-6thC		Kermanshah + Ilam + Lorestan	**3607**
P.012/FN423	2c	Early 5th–mid-6thC	Yes	Kermanshah + Ilam + Lorestan	**3465**
F2.090/FN787	2c	Early 5th–mid-6thC		Kermanshah + Ilam + Lorestan	**3603**
F2.042/FN513	3	Mid-6th–late 7thC	Yes	Khuzestan + Bushehr + Fars	**3459**
F3.015/FN305	3	Mid-6th–late 7thC		Kermanshah + Ilam + Lorestan	**3605**
P.011/FN316	4a	Residual post 7thC	Yes	Kermanshah + Ilam + Lorestan	**3463**
P.011/FN338	4a	Residual post 7thC	Yes	Khuzestan + Bushehr + Fars	**3464**
F2.022/SV182	4a	Residual post 7thC		Khuzestan + Bushehr + Fars	**3602**
F3.007/FN208	4a	Residual post 7thC		Kermanshah + Ilam + Lorestan	**3604**
R.003/FN123	4b	Residual post 7thC		Kermanshah + Ilam + Lorestan	**3608**

**Table 2:** Gross composition of the dichloromethane extract (EO in % by weight/sample) and isotopic data: δ^13^C (‰/VPDB) and δD (‰/VSMOW). Significance of abbreviations: sat = saturated hydrocarbons; aro = aromatic hydrocarbons; NSO = resins; asp = asphaltenes; pol = polars = resins + asphaltenes.

Sample Number	Geomark Reference	EO%/Sample	%sat	%aro	%HC	%NSO	%asp	%pol	δ^13^Csat	δ^13^Caro	δ^13^CNSO	δ^13^Casp	δDNSO Average	δDasp Average
3458	UNK0934	50.5	1.8	2	3.8	9	87.2	96.2	−27.9	−26.8	−26.9	−26.5	−83	−76
3459	UNK0935	39.4	1.7	2	3.7	11.3	85	96.3	−27.7	−26.8	−27	−26.7	−84	−76
3460	UNK0936	43.7	1.9	2.2	4.1	8.2	87.7	96.9	−27.3	−26.6	−26.8	−26.5	−83	−89
3461	UNK0937	40.8	2	2.9	4.9	18.6	76.5	95.1	−27.5	−27.8	−27.1	−26.5	−85	−75
3462	UNK0938	15.1	3.3	6	9.3	37.1	53.6	90.7	−27.3	−27.1	−28.1	−28.1	−97	−91
3463	UNK0939	38.0	1	1.6	2.6	9	88.4	97.4	−29	−28	−28	−27.9	−75	−68
3464	UNK0940	58.9	2.2	1.6	3.8	7	89.2	96.2	−27.6	−26.8	−26.8	−26.3	−80	−79
3465	UNK0941	35.4	1.4	2.6	4	11.7	84.3	96	−28.4	−27.8	−27.9	−27.9	−83	−67
3602	UNK1119	47.8	2.1	2.1	4.2	13.4	82.4	95.8	−27.5	−26.3	−27.1	−26.7	−106	−84
3603	UNK1120	39.6	1.8	1.3	3.1	8.6	88.3	96.9	−28.8	−27.5	−27.6	−27.8	−88	−73
3604	UNK1121	45.6	4.9	3.3	8.2	11.8	80.1	91.8	−29.1	−27.3	−27.2	−27.8	−115	−77
3605	UNK1122	37.3	1.1	1.4	2.5	8.9	88.6	97.5	−28.8	−27.6	−27.9	−27.8	−99	−79
3606	UNK1123	9.8	3.6	3.6	7.2	20.9	71.9	92.8	−27.6	−26.1	−27.1	−26.7	−107	−83
3607	UNK1124	43.7	1.9	1.8	3.7	11.9	84.3	96.3	−28.7	−27.1	−28	−28	−100	−75
3608	UNK1125	23.1	1.2	1.7	2.9	9.5	87.6	97.1	−28.1	−27.6	−28.3	−28	−96	−74

▭ occurrence of oleanane, ▭ no oleanane.

**Table 3:** Molecular data on steranes and terpanes.

Lab Number	GeoMark Number	Site	C30Hppm	Tet/C23	C29/H	OL/H	C31R/H	GA/C31R	GA/H	C35S/C34S	ster/Terp	Dia/Reg	%C27	%C28	%C29	C2920S/R	C29αββ/C29αααR	Ts/Tm	Tricyclics	Terpanes	Steranes	C27diasteranes	c29αααRsterane
**3458**	UNK0934	Fulayj	2001	2.01	0.96	0.13	0.38	0.3	0.11	1.3	0.27	0.72	11.1	30.2	58.7	1.39	1.68	0.52	almost absent	well preserved	biodegraded	present	altered
**3459**	UNK0935	Fulayj	642	1.08	0.93	0.16	0.37	0.44	0.16	1.94	0.37	0.84	10.4	26.4	63.2	1.94	1.6	0.44	almost absent	slightly biodegraded?	biodegraded	present	altered
**3460**	UNK0936	Fulayj	575	1.35	1.02	0.33	0.37	0.41	0.15	2.14	0.65	1.41	11.6	28.1	60.2	2.88	3.95	0.61	almost absent	biodegraded	biodegraded	present	altered
**3461**	UNK0937	Fulayj	157	0.94	1.13	0.27	0.36	0.44	0.16	3.47	0.18	0.87	15.5	26.7	57.8	2.78	1.07	0.46	almost absent	biodegraded	biodegraded	traces	altered
**3462**	UNK0938	Fulayj	1632	1.09	1	0	0.38	0.67	0.25	1.11	0.04	0.33	18.7	23.1	58.2	0.28	0.46	0.14	almost absent	well preserved	preserved	traces	preserved
**3463**	UNK0939	Fulayj	13,716	6.63	0.93	0	0.33	0.63	0.21	0.99	0.06	0.05	19.8	20.6	59.6	0.64	0.94	0.11	almost absent	well preserved	preserved	absent	preserved
**3464**	UNK0940	Fulayj	1321	1.06	0.96	0.15	0.38	0.25	0.09	1.51	0.57	1.26	17	31	52	1.44	2.03	0.6	almost absent	well preserved	slighly biodegraded?	abundant	altered
**3465**	UNK0941	Fulayj	5556	3.57	1.24	0	0.31	0.68	0.21	1.18	0.1	0.15	13.8	23.3	62.9	0.85	1.15	0.19	almost absent	well preserved	preserved?	traces	preserved
**3602**	UNK1119	Fulayj	2536	1.55	0.75	0.14	0.4	0.25	0.1	1.19	0.2	0.88	11.2	29.1	59.8	1.3	1.96	0.5	absent	well preserved	biodegraded	low present	biodegraded
**3603**	UNK1120	Fulayj	11,306	3.21	1.1	0	0.38	0.61	0.23	1.11	0.09	0.08	26.2	24.8	49	0.53	1.04	0.19	almost absent	well preserved	well preserved??	almost absent	preserved
**3604**	UNK1121	Fulayj	2424	1.94	1.18	0	0.32	0.63	0.2	0.9	0.2	1.73	21.4	31.7	46.9	0.73	1.12	0.27	absent	well preserved	preserved	abundant	preserved
**3605**	UNK1122	Fulayj	9480	5.72	1.14	0	0.34	0.66	0.22	1.88	0.07	0.1	14.6	24.2	61.2	0.57	0.86	0.18	absent	well preserved	biodegraded	traces	preserved
**3606**	UNK1123	Fulayj	1617	1.12	0.9	0.12	0.42	0.39	0.16	1.81	0.14	1.21	11.8	23.2	65	1.74	1.71	0.43	absent	well preserved?	biodegraded	present	biodegraded
**3607**	UNK1124	Fulayj	16,149	3.66	1.04	0	0.34	0.56	0.19	0.99	0.07	0.09	24.6	21.7	53.7	0.59	0.94	0.15	almost absent	well preserved	biodegraded?	traces	preserved
**3608**	UNK1125	Fulayj	1723	1.35	1.19	0	0.31	0.9	0.28	2.78	0.13	0.26	6.4	20.1	73.5	1.72	1.58	0.18	almost absent	well preserved?	biodegraded	traces	bioegraded

Significance of abbreviations: C30αβHopane = 17α,21β-hopane, OL/H = 18α(H)-oleanane/hopane, GA/C31αβHR = Gammacerane/17α,21β,22R-30-homohopane, ster/terp = steranes/terpanes, Dia/reg = diasteranes/regular steranes, %C27αββR+S = 5α,14β,17β-20R+20S-cholestane, %C28αββR+S = 5α,14β,17β-20R+20S-24methylcholestane, %C29αββR+S = 5α,14β,17β-20R+20S-24ethylcholestane, C29ααα20S/20R = 5α,14α,17α-20S-24ethylcholestane/5α,14α,17α-20R-24ethylcholestane, C29H/C30H = norhopane/hopane, C27Ts/Tm = 18α-22,29,30-trisnorhopane/17α-22,29,30-trisnorhopane, C35S/C34S = C34-17α,21β-22S-extended hopane/C35- 17α,21β-22S-extended hopane.

**Table 4:** Wine biomarkers detected from archaeological samples. Samples were spiked with a known concentration of Daitzin, which was used as internal standard.

Lab Number	Site	Malvidin 3-Glucoside	Vitisin A	Vitisin B	Daidzin (Internal Standard)
**3458**	Fulayj	absent	absent	absent	present
**3459**	Fulayj	absent	absent	present	present
**3460**	Fulayj	absent	absent	present	present
**3461**	Fulayj	absent	absent	absent	present
**3462**	Fulayj	absent	absent	absent	present
**3463**	Fulayj	present	absent	present	present
**3464**	Fulayj	present	absent	present	present
**3465**	Fulayj	present	absent	present	present

## Data Availability

The original contributions presented in this study are included in the article. Further inquiries can be directed to the corresponding author.
